# Navigating the nexus of social appearance anxiety, learning autonomy, and collaborative dynamics in L2 acquisition: insights from Chinese university L2 learners

**DOI:** 10.3389/fpsyg.2025.1579396

**Published:** 2025-09-05

**Authors:** Weiqi Tian, Jing Liang, Yani Zhao, Mailihaba Aolan, Jingshen Ge

**Affiliations:** ^1^College of Foreign Languages, Xinjiang University, Urumqi, China; ^2^School of Languages, Literacies and Translation, Universiti Sains Malaysia, Penang, Malaysia; ^3^College of Liberal Arts, Journalism and Communication, Ocean University of China, Qingdao, China

**Keywords:** autonomy, collaborative learning orientation, L2 proficiency, structural equation modeling, social appearance anxiety

## Abstract

**Introduction:**

This study examines the interconnected roles of social appearance anxiety, learning autonomy, and collaborative learning orientation in second language acquisition (SLA) among Chinese university students. Using Dynamic Systems Theory as a supplementary theoretical lens, it provides a dynamic interaction perspective for our study.

**Methods:**

Through structural equation modeling, the research identifies the positive impact of learning autonomy and self-regulation on collaborative learning orientation and L2 proficiency though H1 was not supported.

**Results:**

We profoundly discussed this phenomenon within sociocultural context. The study advocates for pedagogical strategies that integrate real-world communication and alleviate social anxieties to enhance learning engagement.

**Discussion:**

Limitations include a cross-sectional approach, limited sampling size (N = 301), limited L2 assessment (CET-6). Prompting recommendations for broader demographic inclusion and refined measurements such as peer review for L2 oral capability etc. The findings stress the need for educational frameworks embracing both formal and informal learning contexts, underscoring autonomy’s pivotal role. This research provides actionable insights for stakeholders to create supportive environments, addressing psychological barriers and emphasizing interactive learning. Ultimately, it contributes to the theoretical advancement and practical improvement of L2 education.

## 1 Introduction

Second language acquisition (SLA) is integral to individual life and cultural exchanges, encompassing diverse domains. As societies become increasingly multilingual and diverse, understanding the complex interactions between language learning, cultural integration, and social dynamics is more important than ever (Pasquarella et al., 2025). SLA is not merely the linguistic equivalent of a native language but is a complex and multi-dimensional field of study (Aprinalistria, 2025).

Research into SLA is a truly multidisciplinary endeavor. Some of the major disciplines that contribute to SLA include theoretical linguistics, education, psychology, and sociology. A growing body of existing research has focused on investigating influencing factors on SLA, such as age (Patkowski, 1980; Pfenninger and Singleton, 2017), aptitude (Granena and Yilmaz, 2018), intelligence (Spolsky, 1989; Taheri et al., 2019), motivation (Lightbown and Anniversary article., 2000; Namaziandost and Rezai, 2024), and personality, all of which have been widely studied. In prior research, these influencing factors have been categorized as cognitive (Zamora et al., 2020) or affective (Portugal-Toro et al., 2025), which can either hinder or facilitate L2 learners’ commitment and enthusiasm to continue learning. The recent emergence of positive psychology within the field of second language acquisition has sparked scholarly interest, while there is still a gap in examining the interplay between diverse stress or anxiety faced by L2 learners, which can influence how these affect regulation (Hanghøj et al., 2022) and L2 learning outcomes.

In this study, we aim to construct a multi-dimensional research model by incorporating social appearance anxiety, self-regulation and collaborative learning orientation collectively into SLA research. The model underscores the necessity of understanding the synergistic interplay among these factors. Expanding the research focus on social appearance anxiety within SLA research not only enriches our understanding of learner dynamics but also necessitates a reassessment of how such socio-emotional variables interact and collectively influence language learning outcomes.

This measure offers significant theoretical and empirical enhancements to the SLA field. The dynamic and intricate nature of second language acquisition involves complex interactions. Within this context, Dynamic Systems Theory (DST), proposed by Dörnyei (1997), has garnered substantial interest. Since the late 1990s, the theory has been applied to second language acquisition (De Bot et al., 2007). Within this theoretical framework, SLA has also been conceptualized as (a) a system of interconnected subsystems; (b) a tendency to self-organization and (c) an occurrence of non-linear (Lowie, 2013), which largely aligns with our research design. We aim to provide a holistic framework that encompasses psychological, affective, and social dimensions of second language acquisition and to investigate the intricate relationship among these constructs, revealing the potential impact of them on L2 achievement. By adopting DST as our supplementary theoretical lens, we further enhancing the depth and breadth of our theoretical framework.

Our cross-disciplinary research aims to make broader contributions to theoretical and empirical understanding in SLA by employing Structural Equation Modeling (SEM), a robust analytical technique suitable for testing complex theoretical models with multiple variables. Additionally, we seek to offer actionable insights for stakeholders, such as educators, educational policymakers, and industry practitioners—to optimize second language learning outcomes in diverse contexts.

## 2 Review of literature

### 2.1 Social appearance anxiety

Social anxiety represents a substantial impairment to daily functioning through its disruption of emotional equilibrium and life dynamics (Tarhan, 2008). This affective state—marked by distress, uncertainty, and perceived loss of control in threat situations (Isik and Pinarcioglu, 1996; Bozdam and Taşğın, 2008) - varies in intensity, particularly during external evaluations of physical appearance. Hart et al. (2008) expand this construct through their conceptualization of social physique anxiety, which transcends simple body image concerns to incorporate wider social perception dimensions.

Within the socio-cultural context, social appearance anxiety refers to a specific type of social anxiety stemming from concerns about others’ evaluations of one’s physical appearance (Yuceant and Unlu, 2017; Esponja et al., 2025). Claes et al. (2012) state that social appearance anxiety as a manifestation of socially expected beauty exacerbates worries by making individuals self-conscious about their bodies potentially leading to social avoidance. Psychological research has extensively investigated the relationships between social appearance anxiety, self-esteem, well-being.

Social appearance anxiety can significantly distort an individual’s body image, fostering negative perceptions of both their physical appearance and their sensitivity to external judgements.

Existing research indicates that social appearance anxiety predominantly focuses on risk factors such as emotional regulation and peer relationships (Zimmer-Gembeck, et al., 2013; Ryding et al., 2025). Alternative approaches could focus on examining the validity or reliability of the Social Appearance Anxiety Scale (SAAS) across different samples, including university students (Doğan, 2010; Levinson and Rodebaugh, 2011; Sahin et al., 2014), adolescents (Blöte et al., 2015), and female patients with eating disorder (Rieger et al., 2010). Alternatively, prior research has primarily focused on symptom presentation and impairments from clinical or general psychology perspective. Scant attention has been paid to general education research, with even less attention to SLA studies. While substantial research have examined key SLA factors, social appearance anxiety, an important psychological construct, remains underexplored in language learning. Scholars like Zimmer-Gembeck et al. (2021) note that moderate appearance-related anxiety commonly occurs in academic environments featuring frequent social interactions, communication, and group learning activities. As established, severe social appearance anxiety adversely affects interpersonal relationships, mental health, and academic performance (Esponja et al., 2025). Notably, as interaction and communication are essential in L2 settings, educators should recognize and address social appearance anxiety during language learning. Creating supportive environments to mitigate its impact and fostering positive social interactions are crucial for both language acquisition and educational success.

L2 acquisition fundamentally requires social interaction for communicative development (Verga and Kotz, 2013). However, many learners experience communication apprehension (Aydin, 2013), frequently due to appearance-related self-confidence issues that hinder classroom participation and learning outcomes. While critically important, the intersection of social appearance anxiety with SLA remains understudied. Our study addresses this gap by examining how such anxiety disrupts language processing (Sellers, 2000; Zheng, 2008), seeking to both elucidate its impacts and advocate for greater consideration in pedagogical approaches.

### 2.2 Collaborative learning orientation

Unlike traditional competitive teaching paradigms, collaborative learning (Kiraly, 2000), emphasizes peer cooperation. Initially proposed by Kiraly (1995), these methodologies, highlighting the need for collective engagement rather than mere task division, necessitating joint efforts toward task completion. This approach enables meaning co-construction and collective cultural knowledge development. By fostering shared learning environments, it enhances L2 educational outcomes through deeper understanding.

Collaborative learning enhances L2 acquisition by promoting cognitive and linguistic development through peer interaction (Kagan, 1995; McGroarty and Scott, 1993). This pedagogical method encourages immersive social interaction in L2 settings, allowing learners to construct knowledge collaboratively. By engaging in small, cooperative learning communities, learners benefit from peer support and active interaction, catalyzing language acquisition. This approach not only facilitates meaningful exchanges but also cultivates a stimulating environment for L2 learners, thereby enhancing their language proficiency through collective efforts and shared experiences.

Kayaoğlu and Sağlamel (2013) examined the dual role of collaborative learning in L2 acquisition, emphasizing both its efficacy in enhancing learning outcomes and its potential to exacerbate language anxiety among learners. While team-based tasks can foster efficient learning by cultivating motivation, responsibility, and positive interrelations—such as commitment, solidarity, and teamwork spirit—these benefits are not universally experienced. Johnson and Johnson et al. (1994) suggest that although such collaborative environments can boost productivity and harmony, they may disadvantage students who struggle with participation or lack self-confidence, leading them to prefer solitary work over group activities. Notably, the intrinsic fear of interaction among some learners can impede their proficiency, highlighting the complex nature of collaboration in education.

In this context, it is critical to recognize that different types of collaboration generate varied outcomes. Educators must be attentive to recognize the individual learners’ differences that might hinder participation. This study anticipates that integrating a supportive classroom community with collaborative learning could serve as a mediating force, balancing the relationship between diverse variables within the research model, thereby creating a more inclusive environment that addresses the potential downsides of collaboration while capitalizing on its advantages.

### 2.3 Autonomy

The concept of learner autonomy in second language (L2) acquisition draws upon a broader psychological construct of “autonomy,” which emphasizes an individual’s independent ability to perform actions and make decisions (Zhang et al., 2022). Within the context of language learning, this notion has garnered significant scholarly interest, leading to various interpretations and definitions of autonomy. Holec (1980) seminally defined learner autonomy as “the ability to take charge of one’s own learning” (p. 3), highlighting self-directed control over the learning process. Expanding further on the concept, Benson (2001) focuses on autonomy’s role in achieving personal learning goals, thereby underscoring its relevance to effective learning outcomes.

Autonomous learners, by definition, exhibit a readiness and willingness to engage with learning activities, which, in turn, enhances their commitment to completing tasks. This intrinsic motivation, a characteristic of autonomous learners, plays a crucial role in facilitating successful language acquisition. Autonomy not only promotes persistence in language learning endeavors but also empowers learners to tailor their educational experiences to fit their individual needs and aspirations.

A nuanced exploration of learner autonomy reveals its multifaceted role as a positive force in language education. Autonomy is intrinsically linked to increased motivation and improved learning outcomes. It allows learners to exercise greater agency over their learning paths which leads to enhanced self-efficacy (Zhou et al., 2023) and resilience against linguistic challenges. Moreover, autonomous learners are more adept at utilizing available resources and strategies to overcome obstacles, thus cultivating a more dynamic and responsive learning environment.

Therefore, learner autonomy should be a central objective in L2 instructional design. Educators can foster autonomy by creating learning environments that encourage self-reflection, decision-making, and goal-setting. Such environments empower learners to take initiative and exercise control over their language education journeys, thereby yielding more meaningful and sustainable learning outcomes.

Contemporary educational research widely recognizes learner autonomy as fundamental for enhancing L2 achievement. Autonomy learning enables proactive engagement and self-directed learning, thereby improving educational outcomes. This study hypothesizes that fostering autonomy in L2 learners holds significantly contributes to language learning success. By enhancing learners’ capacity to initiate and regulate their own learning processes, autonomy serves as a catalyst for attaining higher proficiency and deeper L2 mastery.

### 2.4 Present research

Scholars have investigated L2 learning achievement through key psychological dimensions including emotion regulation, motivation, and learner autonomy (Teimouri, 2017; Csizér et al., 2021), which are crucial for understanding L2 learners’ experiences and their impact on successful language acquisition. In recent years, the focus has expanded to incorporate technological perspectives, reflecting contemporary educational trends. This shift has led to the integration of computer-assisted language learning (CALL) approaches in L2 studies. CALL methodologies facilitate innovative, technology-mediated environments that support personalized learning experiences. By leveraging digital tools, educators can enhance engagement and accessibility, promoting more effective and adaptive language learning outcomes. This evolution in research underlines the importance of harnessing technology to address emerging educational needs and to optimize L2 learning processes within a globalized, digital landscape.

Research in L2 learning has adopted multifaceted perspectives, including the teacher’s viewpoint, which increasingly embraces technologies such as the computer-assisted extended technology acceptance model and artificial intelligence (AI). These tools enhance instructional strategies and learner engagement. Conversely, from the L2 learner’s perspective, however, negative emotional dispositions can impact their engagement, underscoring the importance of addressing emotional factors. These dual perspectives highlight the interplay between technological integration and emotional dynamics, emphasizing the need for balanced approaches that leverage technology while fostering emotionally supportive learning environments conducive to effective language acquisition.

**Figure 1:**
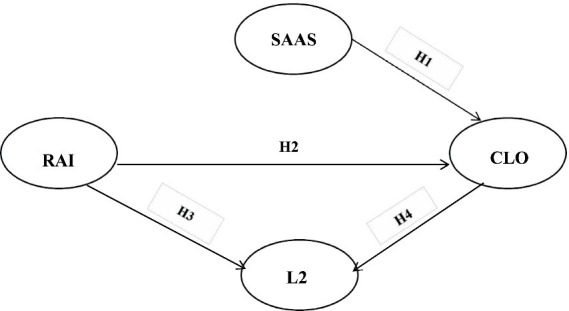
Hypothesized research model.

This study introduces the concept of social appearance anxiety—a relatively unexplored variable in language learning, to investigate its impact on L2 achievement. By integrating this subjective factor with constructs from dynamic systems theory, collaborative learning orientation, and learner autonomy, we examine their collective influence on language learning outcomes. This multifaceted approach aims to elucidate the interconnected relationships between these factors, providing a comprehensive understanding of how socio-psychological dimensions interact to affect L2 proficiency. Such integration offers valuable insights into optimizing instructional strategies for enhanced L2 achievement (Figure 1).

Social appearance anxiety has emerged as a significant research focus, particularly among Chinese youth (Wu et al., 2024). Existing evidence suggests this anxiety negatively impacts academic achievement (Mirawdali et al., 2018). The current study specifically examines its effects on English language proficiency using College English Test Band 6 (CET-6) scores as the outcome measure, aiming to identify psychological barriers to second language acquisition and inform targeted intervention strategies.

In educational environments requiring active participation, appearance-related anxiety may substantially hinder student engagement. As demonstrated by Cowden (2010), heightened social appearance anxiety correlates with reduced participation in collaborative learning activities. Building on this evidence, we argue that such anxiety directly impairs students’ cooperative learning engagement, highlighting the critical need to address psychological factors in creating effective, inclusive learning environments.

*H1:* Social appearance anxiety has a negative effect on collaborative learning orientation.

Extensive research in L2 learning consistently underscores the significant relationship between learner autonomy and language performance. Students with advanced self-regulation skills demonstrate greater English language acquisition (Chen et al., 2022), indicating that autonomous learners are more likely to excel academically (Chirkov and Ryan, 2001; Ryan and Deci, 2006). Such students, capable of effectively monitoring their learning, exhibit heightened enthusiasm and actively engage in collaborative and group tasks. Drawing on this foundation and comprehensive analysis, we posit that fostering autonomy is pivotal for optimizing L2 learning outcomes and academic success.

*H2:* Self-regulation of learning has a positive relation with collaborative learning orientation.

*H3:* Self-regulation of learning has a positive impact on L2 learning performance.

Collaborative learning environments prioritize peer interaction and joint problem-solving, creating dynamic spaces for L2 learners to collaboratively construct language knowledge. Those who engage proactively in such collaboration often experience accelerated progress in language acquisition. Research indicates that collaborative learning exerts a positive and incremental impact on academic achievement (Dörnyei, 1997; Fathman and Kessler, 1992). Based on these insights, we hypothesize that active participation in collaborative learning significantly enhances L2 proficiency, thus serving as a catalyst for improved academic performance in second language contexts.

*H4:* Collaborative learning orientation has a positive effect on L2 learning academic performance.

## 3 Method

### 3.1 Participants and procedure

An initial pool of 314 participants was surveyed online. After the low-quality submissions characterized by abnormal completion times or high repetition responses were excluded, we retained 301 valid responses. As shown in Table 1, the sample comprised52 males (17.3%) and 249 females (82.7%) from various Chinese universities. Notably, 92.7% of participants chose English as their foreign language, spanning from freshman to senior year. A majority, 59.5%, had passed the CET-6 exam, which is a widely recognized benchmark for English proficiency in China. This demographic composition provides a robust foundation for analyzing factors influencing L2 acquisition across diverse student groups within the Chinese higher education context.

**Table 1:** Demographical characteristics of participants (*N* = 301).

Characteristic	Category	Frequency	Percentage
Gender	Male	52	17.3%
	Female	249	82.7%
EFL	Yes	279	92.7%
	No	22	7.3%
Passed the CET-6	Yes	179	59.5%
	No	122	41.5%
Grade	Grade 1	9	2.9%
	Grade 2	58	19.3%
	Grade 3	113	37.5%
	Grade 4	121	40.3%

This study adhered to ethical guidelines, ensuring anonymous and voluntary participation. Informed consent was obtained prior to data collection, and all responses were kept confidential. All respondents could withdraw at any time without incurring adverse consequence.

### 3.2 Measures

#### 3.2.1 Social appearance anxiety

The Social Appearance Anxiety Scale (SAAS) (Hart et al., 2008), is a psychometric tool designed to evaluate levels of social appearance anxiety. Initial validation showed excellent internal consistency, test-retest reliability, and convergent validity (Levinson and Rodebaugh, 2011). The 16-item scale includes statements like “I get nervous when talking to people because of the way I look,” and one reverse-coded item “I feel comfortable with the way I appear to others.” Responses use a 5-point Likert scale (1 = not at all to 5 = strongly agree), with higher scores indicating greater anxiety. The SAAS effectively measures appearance-related anxiety and its psychological impacts across various contexts. In this study, it demonstrates satisfactory consistency (Cronbach’s alpha = 0.882).

#### 3.2.2 Autonomy

Our study adapted Ryan and Connell’s (1989) Academic Self-Regulation (SQR-A) Questionnaire to assess the regulatory styles of L2 learners. Self-reported SQR-A has been found to predict academic achievement and discrimination between high/low achievers (Pierson and Connell, 1992; Pintrich, 2000; Winne and Perry, 2000), which also has been applied in the existing studies, showing adequate validity and internal consistency (Zamora et al., 2020; Vansteenkiste et al., 2012; Vergara-Morales and Del Valle, 2021; Vergara-Morales et al., 2019; Vergara-Morales et al., 2022).

This 22-itemrevised version includes statements like, “I do my English homework so that my parents and teacher won’t be mad at me.” Participants rated on a 5-point scale (totally disagree to totally agree) and Higher scores on this scale denote greater learner autonomy in language learning, highlighting self-directed motivation. This instrument thus provides insights into the degree of self-regulation among L2 learners and its implications for their educational outcomes, offering a framework for understanding motivational influences on language acquisition within diverse learner populations. Our scale has shown high reliability (Cronbach’s alpha = 0.857).

#### 3.2.3 Collaborative learning orientation

This study utilized selected components of the Collaborative Learning Attitude Scale (CLAS) (Haidari and Uzun, 2019). The scale encompasses multiple dimensions, including self-efficacy in EFL, language proficiency, self-regulation in EFL, motivation in EFL, and affective attitudes, providing a comprehensive measure of interactive dynamics in educational contexts to align with the focus on collaborative learning, we specifically selected the Affective Attitudes subscale of the CLAS. This targeted approach served dual purposes: (a) the subscale items aligned with our research purpose of examining the participants’ inclincation toward collaborative learning (e.g., ‘I enjoy working with peers’); and (b) the length of the subscale (8-item) maintained methodological rigor while optimizing respondent burden during survey administration.

The initial version of CLAS has been subjected to three rounds of expert reviews and demonstrates a considerable internal consistency, with Cronbach’s alpha values for the overall scale and subscales ranging between 0.68 and 0.87 (Haidari and Uzun, 2019). Fourteen items were adapted from the original scale, with modifications to optimize our study. Example items include: (“Collaborative learning with peers can make English classes more engaging”); “Practicing English with peers makes me feel relaxed.”

A five-point Likert scale (1 = Strongly Disagree to 5 = Strongly Agree) was employed.

Higher scores indicate a stronger inclination toward peer engagement, which correlates positively with improved L2 learning outcomes. This instrument enables systematic examination of how L2 learners’ collaborative learning willingness may influence their L2 achievement, while assessing the whether differential intention toward collaboration may affect L2 proficiency or not. In this study, our Cronbach’s alpha for reliability was 0.788.

#### 3.2.4 L2 achievement

In evaluating participants’ L2 proficiency, the College English Test-6 (CET-6), which is an English testing system for Chinese undergraduates and postgraduates, has been employed as a definitive standard. Administered by the National College English Testing Committee, it is regarded as an impartial and accurate measure of the English proficiency of college and university students in China and plays an indispensable role in evaluating students’ English language skills, including listening, reading, writing, and speaking (Yan et al., 2024). Within China’s College English Test (CET) framework, a score of 425 has been universally served as a passing threshold for both CET-4 and CET-6. The CET testing system has given colleges nationwide in China a uniform standard of comparison on the efficacy of English language teaching and learning (Zheng and Cheng, 2008; Yan et al., 2024). Building on this, we adopted the passing threshold (≥425 points) as a representation of L2 achievement. The aforementioned content enables an assessment of both receptive and expressive language abilities. In this study, participants passed the CET-6 reached at 59.5% (*N* = 179).

However, it’s necessary to acknowledge that there are certain inherent limitations in the CET-6. For instance, a defining characteristic of the CET-6 is exam-oriented rather than keeping the focus on practical communication and interaction.

### 3.3 Data analysis

Our analytical approach comprised multiple statistical methods to examine the relationships among the variables involved in the study. First, descriptive statistics and Pearson’s correlation analyses were conducted by using SPSS Statistics 27.0. Subsequently, we adopted structural equation modeling (SEM) using Smart PLS 4.0 to test the internal reliability and validity of our hypothesized model through some indicators such as factor loadings of each item, KMO values of each sub-scale. Convergent validity and discriminant validity were also been checked through observations of values of construct reliability (CR), average variance extracted (AVE) and heterotrait-monotrait ratio of correlations (HTMT). In the final phase, bootstrap analysis with 5,000 iterations was performed, some model fit indices and their criteria were employed to examine the goodness of model fit with the given indicators: standardized root mean square residual (SRMR) and normed fit index (NFI). Our hypotheses (H1–H4) were verified through significance testing of path coefficients and *p*-values, and the mediation effects were examined through indirect path analysis in L2 context.

## 4 Results

### 4.1 Descriptive analysis and correlational analysis

Table 2 presents the descriptive statistics, including means, standard deviations, and results from the normality tests of the sample data. Participants showed no significant differences across the SAAS, Academic Self-Regulation Questionnaire (SQR-A), and Collaborative Learning Attitude Scale, with relatively low SAAS scores. The skewness and kurtosis measures were within the acceptable range of ± 2.0 (Kim, 2013; Zarrinabadi et al., 2024), indicating a near-normal distribution of the data. Additionally, the reliability of the composite questionnaire was satisfactory, as Cronbach’s *α* for all scales exceeded the threshold of 0.7, as suggested by Cheng and Dörnyei (2007).

**Table 2:** Descriptive results for SAA, RAI, CLO.

Variable	Mean	SD	Skewness	Kurtosis
Kurtosis SAA	3.061	0.859	−0.149	−0.983
RAI	3.351	0.309	−0.114	0.251
CLO	3.509	0.458	−0.376	0.118
ACHI	0.59	0.492	−0.388	−1.862

*N* = 301. SAA, Social Appearance Anxiety; RAI, Relative Autonomy Index; CLO, Collaborative Learning Orientation; ACHI, English Achievement.

Bivariate correlation analysis using SPSS Statistics 27 revealed that L2 learning autonomy positively correlates with the willingness for collaborative learning (refer to Table 2). Conversely, variables such as Regulatory Autonomy Index (RAI), Collaborative Learning Orientation (CLO), and Academic Achievement (ACHI) were negatively correlated with Social Appearance Anxiety (SAA). A stronger negative correlation was found between SAA and both RAI and CLO. Notably, RAI and CLO demonstrated high inter-correlation, suggesting that RAI act as a positive motivator, enhancing the orientation toward collaborative learning. This dynamic underscores the potential of regulatory autonomy to foster collaborative learning environments, which are crucial for effective language learning.

The existing body of literature extensively examines gender differences (Zhou, 2016); however, due to the substantial gender imbalance in our sample (249 females vs. 52 males), a gender difference analysis was deemed inappropriate. Descriptive statistical analysis revealed a progressive increase in the CET-6 passing rates across academic grades—11, 34.5, 64.6, and 70.2%, respectively. This observation prompted the application of a Pearson correlation test to investigate potential intricate relationships among variables across different undergraduate levels. By focusing on grade-based variations in CET-6 performance, this study aims to uncover complex interdependencies in language proficiency that may be influenced by academic progression, offering insights into the evolving nature of language acquisition. Such an approach provides a nuanced understanding of the factors that drive academic success in L2 learning environments across varying educational stages.

As illustrated in Table 3, social appearance anxiety negatively impacts both self-regulation in English learning and orientation toward collaborative learning, statistically significant but weak negative correlations were observed (shown, respectively, *r* = −0.223, *p*<0.01; *r* = −0.177, *p*<0.01). Concurrently, the data reveal a robust positive correlation between the Regulatory Autonomy Index (RAI) and Collaborative Learning Orientation (CLO), particularly as the number of participants passing the CET-6 exam increases with academic progression (shown in Table 4). Notably, among fourth-year university students, a strong positive relationship exists between RAI and English learning achievement. These findings suggest that while social appearance anxiety can hinder learning autonomy and group learning tendencies, its influence relatively mild compared to other factors.

**Table 3:** Correlations between SAA, RAI, CLO, ACHI.

Item	SAA	RAI	CLO	ACHI
SAA	1			
RAI	−0.223**	1		
CLO	−0.177**	0.479**	1	
ACHI	−0.037	0.083	0.130*	1

*N* = 301, ***p<0.001, **p<0.01, *p<0.05 (2-tailed).

**Table 4:** Bivariate correlations across different grades.

Item	SAA	RAI	CLO	ACHI
Grade 1				
SAA	1			
RAI	0.08	1		
CLO	−0.331	0.596	1	
ACHI	−0.636	−0.091	0.27	1
Grade 2				
SAA	1			
RAI	−0.382**	1		
CLO	−0.274**	0.507**	1	
ACHI	−0.207	0.18	0.147	1
Grade 3				
SAA	1			
RAI	−0.239*	1		
CLO	−0.11	0.382**	1	
ACHI	−0.033	−0.054	0.072	1
Grade 4				
SAA	1			
RAI	−0.14	1		
CLO	0.181*	0.569**	1	
ACHI	0.03	0.272**	0.149	1

*N* = 301, ***p<0.001, **p<0.01, *p<0.05 (2-tailed).

Importantly, across all undergraduate levels, a consistent positive association between RAI and CLO emerges. This finding underscores the pivotal role of self-regulation in foreign language learning, which exerts a more substantial effect on English learning outcomes than does social appearance anxiety. The results suggest that developing self-regulation skills could substantially enhance academic achievement in L2 contexts, thereby offering valuable insights for educators aiming to improve language proficiency among students across diverse educational stages.

### 4.2 Structural equation model

Prior to structural equation modeling analysis, all observed variables were rigorously evaluated for reliability, convergent validity and discriminant validity using Smart PLS 4.0. This verification process confirmed that the empirical relationships between items and their corresponding constructs aligned with theoretical expectations. Factor loadings for each item were calculated to verify these relationships. Following established methodologies (Tabachnick and Fidell, 2007; Comrey and Lee, 2013, Hair Jnr et al., 2010), items with factor loadings below 0.5 were removed to enhance model reliability. Building on this criterion, the following items were removed: (a) Social Appearance Anxiety Scale: Items 1–3, 5–7, 9, 11, 13, 15; (b) Academic Self-Regulation Scale: Items 1–2, 4, 6, 8–9, 11–12, 14–16, 18, 21–22; and (c) Collaborative Learning Attitude Scale: Items 3–4, 7, 9–10, 13–14 (see Table 5).

**Table 5:** Factor loadings and validity of scales.

Scale	Item	Factor loadings	KMO value
SAAS	SAAS4	0.617	
	SAAS8	0.703	
	SAAS10	0.784	0.872
	SAAS12	0.782	
	SAAS14	0.879	
	SAAS16	0.742	
SR	SR3	0.695	
	SR5	0.696	
	SR7	0.744	
	SR10	0.688	
	SR13	0.674	0.897
	SR17	0.810	
	SR19	0.681	
	SR20	0.664	
CLO	CLO1	0.609	
	CLO2	0.724	
	CLO5	0.724	
	CLO6	0.584	0.842
	CLO8	0.719	
	CLO11	0.684	
	CLO12	0.574	

*N* = 301. SAAS, Social Appearance Anxiety Scale; SR, Self-Regulation of English learning; CLO, Collaborative Learning Orientation.

The low factor loadings could be attributed to two reasons. First, during the process of back translation, the phrasing of the items contains subtle bias, which may result in ambiguities in understanding; second, it is plausible that items may measure constructs other than the intended factors.

The refined model demonstrates satisfactory psychometric properties, with all factor loadings and Kaiser-Meyer-Olkin (KMO) values falling within acceptable ranges (SAAS = 0.872, ASR-Q = 0.897, ClAS = 0.842), consistent with established criteria (Field et al., 2009; Guttman, 1954; Kaiser, 1970; Shrestha, 2021) where KMO values between 0.8 to 1.0 indicating the sampling is adequate, which validated the model’s structure and also indicated our revisions of sub-scale is acceptable. This rigorous evaluation underscores the methodological robustness of the analysis, ensuring that the theoretical constructs are adequately represented by the measurement model. Addressing these methodological aspects enriches our understanding of complex constructs in the study, thus advancing academic discourse in L2 learning research.

Table 6 presents the Cronbach’s *α* values for each scale. All subscales demonstrated strong internal consistency, with Cronbach’s α values exceeding the 0.70 threshold (SAAS = 0.882, ASR-Q = 0.857, CLAS = 0.788), meeting the minimum reliability standard proposed.

**Table 6:** The reliability, validity, and discriminant validity of each observed variable.

Variable	SAA	RAI	CLO	α	Composite reliability (rho_a)	Composite reliability (rho_b)	AVE
SAA			0.128	0.882	0.786	0.887	0.571
RAI	0.081		0.658	0.857	0.86	0.889	0.501
CLO				0.788	0.799	0.844	0.439

*N* = 301. SAAS, Social Appearance Anxiety; RAI, Relative Autonomy Index; CLO, Collaborative Learning Orientation. The discriminant validity of each observed variable is their respective HTMT values.

In addition, composite reliability (CR) scores further supported the model’s robustness ranged from 0.844 to 0.889. Underscoring the reliability of the hypothesized model. Besides Collaborative Learning Orientation (AVE = 0.439), both the Social Appearance Anxiety and Regulatory Autonomy Index scales exhibit average variance extracted (AVE) values exceeding 0.5 (ranged from 0.501 to 0.571), alongside VIF values remaining below 5. This indicates stability in structural variables and strong convergent validity.

Furthermore, Heterotrait-Monotrait (HTMT) ratios confirmed minimal collinearity among variables, substantiating the model’s suitability for SEM analysis (Hair et al., 2019). Together with the previously established reliability and validity metrics, these results comprehensively validate the model’s robustness in capturing the complex interrelationships among the studied variables.

**Figure 2:**
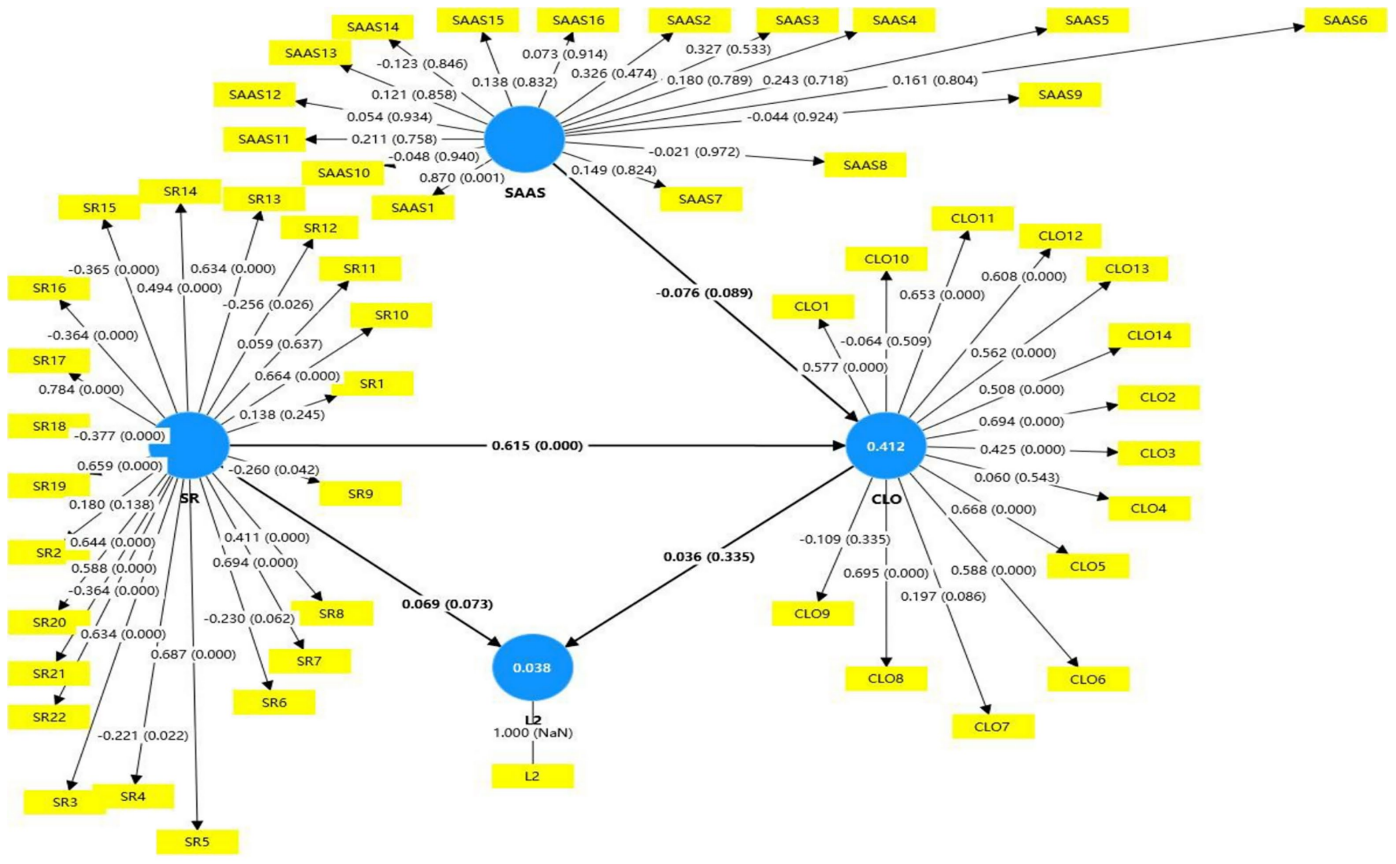
Structural equation modeling of hypothesized model. *N =* 301. SAAS, Social Appearance Anxiety Scale; SR, Self-Regulation of English learning; CLO, Collaborative Learning Orientation; L2: Achievement of CET-6. The path coefficients are standardized.

Following initial confirmation of the research model’s suitability for SEM analysis, preliminary evaluations revealed inadequate fit indices (SRMR = 0.18, NFI = 0.245), falling substantially below acceptable thresholds (see Figure 2). These results necessitated significant model modifications.

**Figure 3:**
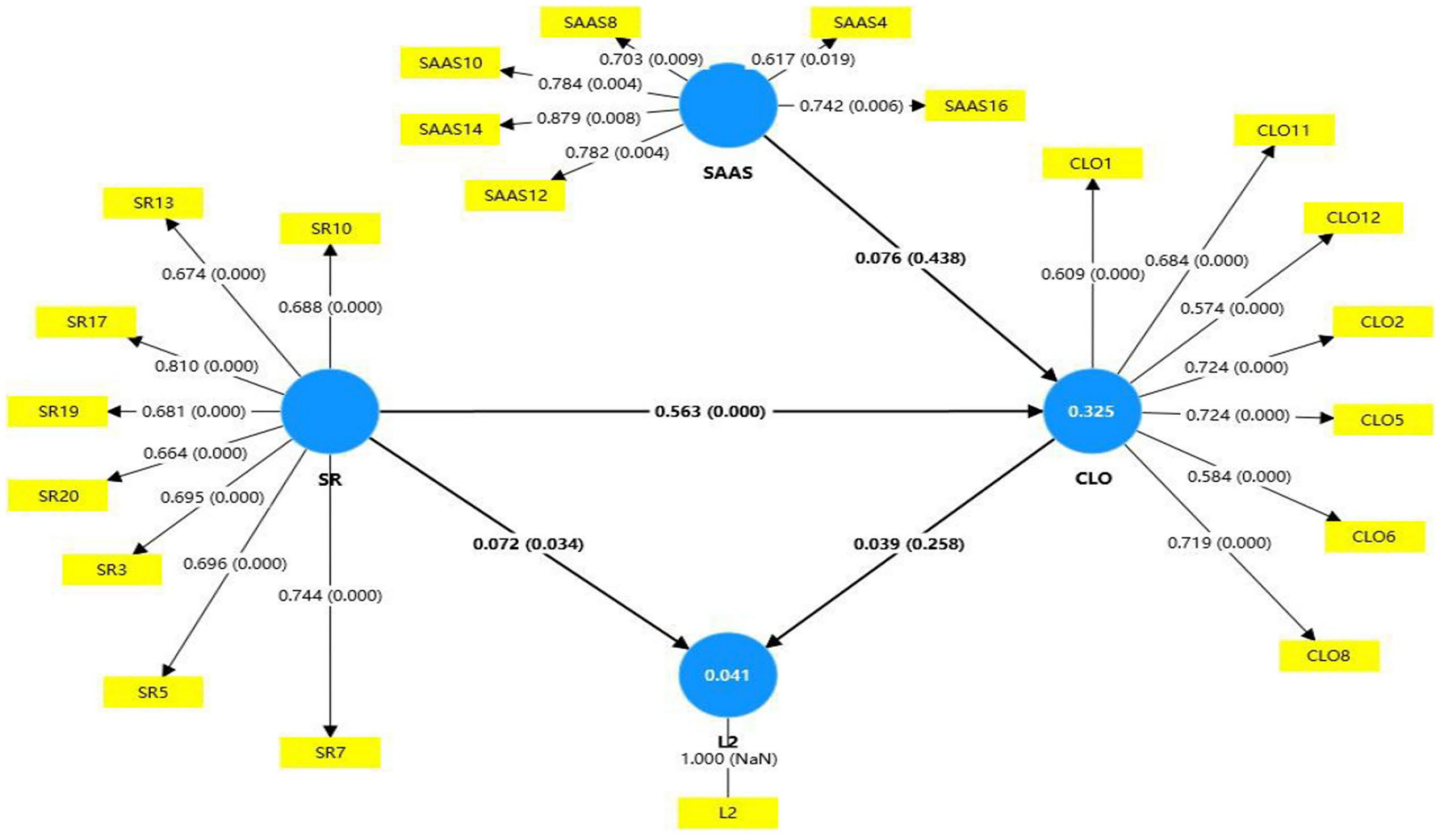
Structural equation modeling of corrected model. *N =* 301. SAAS, Social Appearance Anxiety Scale; SR, Self-Regulation of English learning; CLO, Collaborative Learning Orientation; L2: Achievement of CET-6. The path coefficients are standardized.

Subsequently, based on the refined research model after removing items with substandard factor loadings, we contined to conduct a model fit assessment. In the second round of evaluation, an acceptable fit was achieved, evidenced by improved indices: *χ*^2^ = 498.259, SRMR = 0.067, NFI = 0.804 (see Figure 3). These modifications underscore the iterative nature of SEM, emphasizing the importance of continuous model assessment.

The improved model fit enhances the reliability of the subsequent analyses, providing a more accurate framework for exploring the intricate dynamics of L2 learning processes. This methodological rigor ensures the validity of conclusions drawn from the SEM, contributing valuable insights to the academic discourse on language acquisition.

In subsequent analysis, path coefficients and *p*-values were calculated using the path coefficient diagram, with a bootstrap procedure of 5,000 samples informing the SEM results (see Figure 2). Within the revised model, the Regulatory Autonomy Index (RAI) emerged as a significant positive predictor of Collaborative Learning Orientation (CLO) with a path coefficient of *β* = 0.563, *p* = 0.000. It also positively influenced English learning achievement, although this relationship was weaker (*β* = 0.072, *p* = 0.034) compared to RAI’s effect on CLO.

Other hypothesized relationships, such as SAAS→CLO (*β* = 0.076, *p* = 0.438) and CLO → L2 (*β* = 0.039, *p* = 0.258), lacked statistical significance. Furthermore, there was no evidence of mediation among Social Appearance Anxiety (SAA), CLO, and L2 learning (*p* = 0.592, with confidence intervals including 0). Similarly, CLO did not mediate the relationship between RAI and L2 acquisition (*p* = 0.276, with confidence intervals including 0).

Nevertheless, the direct effects of RAI on both CLO and L2 learning achieved statistical significance, emphasizing RAI’s role as a critical determinant of collaborative and academic outcomes. These findings highlight the pivotal influence of regulatory autonomy in fostering effective collaborative learning environments, ultimately enriching language acquisition processes (Table 7).

**Table 7:** Direct and indirect path coefficients of the hypothesis model.

				97% confidence interval	
Research path	SD	*t*	*p*	Lower	Upper
CLO → L2	0.034	1.131	0.258	−0.027	0.108
SAA → CLO	0.099	0.775	0.438	−0.196	0.159
SAA → L2	0.006	0.536	0.592	−0.008	0.015
RAI → CLO	0.043	13.096	0	0.465	0.636
RAI → L2	0.028	3.354	0.001	0.036	0.147
SAA → CLO → L2	0.006	0.536	0.592	−0.008	0.015
RAI → CLO → L2	0.02	1.09	0.276	−0.015	0.065

*N* = 301. SAAS, Social Appearance Anxiety; RAI, Relative Autonomy Index; CLO, Collaborative Learning Orientation. L2, Achievement of CET-6.

## 5 Discussion

Our findings generally supported the hypotheses, revealing expected bivariate correlations. Social appearance anxiety negatively correlated with learning autonomy and collaborative learning orientation, both of which shared a robust positive association. These results conform to previous studies showing that social appearance anxiety, as a form of social evaluation anxiety, negatively impacted social evaluation anxiety and self-regulation (Zhong et al., 2024). Within sociocultural context, social appearance anxiety may be exacerbated by factors, including social comparison occurred in social media (Papapanou et al., 2023; Baltaci et al., 2021) and social evaluative concerns regarding physical appearance.

Analyzing samples by undergraduate levels, it emerged that collaborative learning orientation consistently related to learning autonomy across all groups. This finding corroborates existing literature suggesting that autonomous learners are more inclined to pursue and benefit from collaborative educational settings (Ames, 1992; Benson, 2013; Summers et al., 2009).

For freshmen and sophomores engaged in our survey, social appearance anxiety impacted their participation in group activities and class discussions, potentially diminishing their English learning outcomes (percentage of CET-6’s passing rate only 11 and 35%). This emphasizes the need for interventions targeting anxiety reduction to enhance these students’ engagement and performance. Conversely, senior students demonstrated a higher propensity for effective self-regulation in their learning processes. This capability allowed them to manage their foreign language studies more proficiently, organize study schedules, and monitor learning progress, thereby providing substantial support in their efforts for better language achievements (r between self-regulation and L2 achievement = 0.272, *p* ≤ 0.01). These insights highlight the evolving influence of psychological and regulatory factors on language learning across different stages of academic development, offering targeted opportunities for educational interventions to enhance L2 learning outcomes.

In contrast to their junior counterparts, grade 3 college students exhibit resilience against the impacts of social appearance anxiety, coinciding with a marked improvement in CET-6 passing rates, a key measure of L2 learning outcomes. This phenomenon may exhibit a tentative inverse association with the detrimental effects of social appearance anxiety on L2 achievement and collaborative learning orientation. (r value between SAAS and CLAS changed from −0.331 to −0.11 and r value between SAAS and L2 achievement changed from −0.636 to −0.033). As students advance in their college years, they experience an enrichment of language knowledge, facilitated by continuous learning and cognitive development, which collectively enable a more robust academic foundation. This dynamic revealed that social appearance anxiety exerts a certain degree of influence on L2 learners’ affective attitude and academic performance, which agreed with past research conducted by Chirkov and Ryan (2001) and Ryan and Deci (2006).

Further verification of our assumptions was achieved through SEM, which confirmed several hypotheses, notably that self-regulation positively influences collaborative learning orientation (H2). This finding aligns with prevailing academic perspectives, suggesting that students with higher autonomy are more likely to actively participate in diverse learning activities and exhibit greater engagement (Zhou, 2016). Such autonomy facilitates a proactive approach to learning, fostering an environment conducive to academic collaboration and enriched educational experiences. This underscores the pivotal role of self-regulation in enhancing both individual and collective learning dynamics.

As verified by Tomak and Seferoglu (2021) L2 learners with high levels of self-regulation will demonstrate better English verbal skills and written performance, indicating a direct and positive connection between self-regulation and L2 proficiency, which underscores why learning autonomy positively influences L2 learning outcomes (H3). This conclusion is firmly established in the extant literature (Pintrich and De Groot., 1990; Labuhn et al., 2010; Mahmoodi et al., 2014). A body of research confirmed that self-regulation enhances motivational factors, even exhibiting a reciprocal relationship between self-regulation and other motivational elements such as intrinsic value, self-efficacy (e.g., Guo et al., 2023; Teng et al., 2024). In light of these findings, a new insight aimed at examining the interplay between self-regulation and collaborative learning was provided in our study. There is a potential positive association (r = 0.479, *p* ≤ 0.01) between them, with mutual predictive power (*β* = 0.563, *p* = 0.000), this observation also agreed with relevant conclusions (Ucan and Webb, 2015; Ucan, 2017), which claimed that Regulation of learning is critically connected with collaborative learning, shown in several aspect as: promote knowledge construction, advance the process of learning and enhance the quality and efficiency of collaborative learning. Nevertheless, two hypotheses remain unverified (H1 and H4). Distinct from studies involving social anxiety or participants in K-12 education, the CET-6 score showed only a subtle relationship with collaboration aversion linked to appearance anxiety. Although CET-6 exerts great influence in higher education domains, as evidenced by the past research as “since the early 1990s, CET-4/6 was classified as a mandatory test by most higher education institutions. For non-English major college students, it was even a precondition for them to obtain the degree diplomas and seek personal gains (Jiang, 2020).” This test is still featured as exam-oriented assessments. Scores of CET-4/6 may not adequately capture a learner’s expressive or interactive capabilities, thus failing to convey the holistic practicality inherent in mastering a second language.

Individuals’ social appearance anxiety may exert a detrimental impact on their social interaction. Given that no collaborative learning tasks were assigned by teachers but it is more dependent on L2 learners’ academic practices and self-assessments, thus the negative effect of social appearance anxiety may be mitigated in preparation of CET-6. Above-mentioned assumption provides an account against Hypothesis 1.

The analysis revealed no significant indirect effect of learning autonomy on L2 outcomes through collaborative learning orientation, nor between social appearance anxiety and L2 results. This aligns with earlier research suggesting that the weak link between collaboration willingness and L2 proficiency may stem from social appearance anxiety’s negative impact (Zhou, 2016). These findings underscore the complexity of social factors in language acquisition, highlighting the need to further explore how psychological barriers affect learning dynamics and outcomes.

The Academic Self-Regulation Questionnaire, developed under the framework of self-determination theory, divides the autonomy scale into four components: intrinsic motivation, identified regulation, introjected regulation, and external regulation. The revised model predominantly utilizes items from intrinsic motivation (e.g., “I engage in English assignments because I find the process of learning English intrinsically interesting”) and identified regulation (e.g., “I complete English assignments because I want to master English language”). Intrinsic motivation emphasizes inner drives like interest and satisfaction, encouraging active L2 learning and stimulating enthusiasm for acquiring a second language (Ames, 1992; Benson, 2013; Summers et al., 2009). In contrast, identified regulation focuses on enhancing learners’ awareness of L2 importance and fostering self-initiative, prompting them to engage in learning activities rather than under external pressures. These insights highlight the significance of nurturing internal motivations and awareness to promote sustainable L2 learning engagement.

The analysis underscores the critical role of learning autonomy, particularly intrinsic motivation and identified regulation, in enhancing academic outcomes. For university students, fostering greater autonomy is essential. This involves cultivating intrinsic motivation and a clear understanding of language learning’s importance, enabling students to engage more deeply and independently in their educational pursuits. This aligns with self-determination theory, promoting sustainable and self-motivated learning behaviors.

## 6 Conclusion

In conclusion, this study aimed at further advancing L2 research by constructing and validating a novel interdisciplinary hypothesized model that integrates social appearance anxiety (psychological factor), self-regulation (metacognitive element), and collaborative learning orientation (social-affective dimension). The findings empirically examined how these constructs collectively influence L2 achievement and revealed their dynamic interactions. Notably, the innovative inclusion of social appearance anxiety extends theoretical boundaries in SLA contexts, given the limited scholarly engagement with its role in second language acquisition.

Our results confirmed the well-established conclusion that autonomy exerts a positive relationship on L2 achievement, aligning with prior research. However, we drew a conclusion from our hypothesis that collaborative learning orientation failed to emerge as a positive predictor of L2 proficiency, which is contrary to predominant SLA literatures. This phenomenon may stem from several contributing factors, such as social phobia, physical appearance anxiety, personality traits or any other factors. Additionally, our findings suggest that collaborative learning does not invariably appear to be effective and efficient. Building on existing literature and public discourse, social appearance anxiety may adversely affect numerous aspects, such as collaborative learning dynamics, social interaction pattern, and L2 learning proficiency, particularly oral capability. In our results, we revealed that this negative impact may be interpreted within specific research frameworks. Our study also yields indispensable contributions from both theoretical and practical aspects in SLA research field.

From theoretical perspective, our study drew upon Dynamic System Theory adopted by Dörnyei (2009) as a critical supplementary theoretical lens. In terms of research methodology, this study not only enhances the applicability of the dynamic system theory in SLA but also extends its theoretical scope by incorporating constructs into a holistic research model. Furthermore, this study extends the demographic paradigm beyond K-12 populations (elementary and secondary students), adolescents, to incorporate college students thus providing some pedagogical insights for tutors and education authorities. Moreover, this study also provides new directions for future SLA research in several aspects: demographic diversification, interdisciplinary synergy and dynamic methodologies.

### 6.1 Limitations and implications

While our study provides novel insights into SLA, several limitations should be noted: (a) Cross-sectional limitation. Despite employing dynamic system theory as our complementary theory, our analytical approach still be characterized by cross-sectional, demonstrating lack of and the temporal depth of longitudinal studies. Cross-sectional design may preclude causal inferences and self-report questionnaire may introduce bias (Aldoais et al., 2025). In the process of examining L2 and its dynamic relationship, some influencing such as personality traits or L2 grit of participants even will be omitted within the given hypothesized model. (b) Non-representative sampling. Notwithstanding our sampling size (*N* = 301) is basically acceptable, significant gender disparity exists (82.7% female and 19.3% male), which may result in limited generalizability of validation of our hypotheses across gender. (c) Non-representative assessment criterion of L2 achievement. Although CET-6 is regarded as a way for measuring L2 proficiency of undergraduates and postgraduates in universities, we have to acknowledge that it is not applicable to assess L2 learners’ oral capability. To address these limitations, future research should incorporate: (a) Adopt a longitudinal design to further investigate the dynamic changes of L2 learning. Subsequently, diverse research methods and tools can be combined so as to enhance the generality of our findings, for instance, questionnaire survey + semi-structured interview. (b) Enlarge our sampling size or employ stratified sampling so as to increase the precision and reliability of our finding. (c) Enrich the assessment standards of L2, such as in-class performance and peer-review or some evaluation of L2 academic tasks assigned by teachers.

Despite inherent limitations, this study offers valuable insights and recommendations for various stakeholders. School authorities, in particular, should provide financial and infrastructural support to educators, enabling the implementation of diverse pedagogical activities designed to ignite students’ interest in English learning. Sufficient funding support may promote R and D personnel to develop more advanced teaching and learning applications, which allows students to have access to much more learning resources via online platforms thus enhancing their learning autonomy of second language (Liu et al., 2024).

Tutors are pivotal in fostering an immersive learning environment where students can fully engage with educational activities. Educators should prioritize ample encouragement and recognition, which aids in building students’ self-confidence while alleviating foreign language learning anxiety. As suggested by Mariani (1997), greater use of positive reinforcement and lenient evaluation of student responses promote more active classroom participation, encouraging learners to engage enthusiastically without fear of judgment and ultimately enhancing their academic performance and L2 learning enthusiasm. Furthermore, engaging teaching activities should be conducted so as to strengthen L2 learners’ collaboration, for instance, conducting scenario-based tasks or setting open-ended questions to enhance collaborative discussion among students, which may enhance students’ tendency toward collaborative learning. Anonymous response is also encouraged, which may make it feasible to alleviate the effect of social appearance anxiety of L2 learners on L2 learning.

For L2 learners, building self-confidence and developing positive self-regulation skills can mitigate the impact of social appearance anxiety on L2 learning. Additionally, they should also be encouraged to make better use of diverse online or offline L2 learning materials to strengthen self-regulation of L2 learning. Meanwhile, L2 learners should recognize the benefits of effective collaboration for L2 acquisition and actively engage in collaborative learning activities.

In contemporary society, where communication and interaction are paramount, overemphasis on individuals’ appearance or body image is unproductive. Social appearance anxiety remains a significant concern, particularly for L2 learners. Reducing such anxiety is crucial, as it can substantially enhance learners’ language performance. Fostering an environment that values substance over superficial judgments helps alleviate anxiety, thereby facilitating more effective language acquisition and communication skills (Weiping and Juan, 2005). Addressing these issues is vital to creating supportive, inclusive educational settings that empower learners to excel linguistically.

## Data Availability

The raw data supporting the conclusions of this article will be made available by the authors, without undue reservation.
